# Time-Dependent Fluid-Structure Interaction Simulations of a Simplified Human Soft Palate

**DOI:** 10.3390/bioengineering10111313

**Published:** 2023-11-14

**Authors:** Peng Li, Marco Laudato, Mihai Mihaescu

**Affiliations:** Department of Engineering Mechanics, FLOW, KTH Royal Institute of Technology, 10044 Stockholm, Sweden; mihaescu@kth.se

**Keywords:** obstructive sleep apnea, fluid-structure interaction, soft palate, 3D simplified model

## Abstract

Obstructive Sleep Apnea Syndrome (OSAS) is a common sleep-related disorder. It is characterized by recurrent partial or total collapse of pharyngeal upper airway accompanied by induced vibrations of the soft tissues (e.g., soft palate). The knowledge of the tissue behavior subject to a particular airflow is relevant for realistic clinic applications. However, in-vivo measurements are usually impractical. The goal of the present study is to develop a 3D fluid-structure interaction model for the human uvulopalatal system relevant to OSA based on simplified geometries under physiological conditions. Numerical simulations are performed to assess the influence of the different breathing conditions on the vibrational dynamics of the flexible structure. Meanwhile, the fluid patterns are investigated for the coupled fluid-structure system as well. Increasing the respiratory flow rate is shown to induce larger structural deformation. Vortex shedding induced resonance is not observed due to the large discrepancy between the flow oscillatory frequency and the natural frequency of the structure. The large deformation for symmetric breathing case under intensive respiration is mainly because of the positive feedback from the pressure differences on the top and the bottom surfaces of the structure.

## 1. Introduction

Obstructive Sleep Apnea (OSA) is a common, complex and highly prevalent sleep-disordered breathing condition [1], which is characterized by partial or complete cessation of airflow during sleep owing to upper airway collapse [2]. Estimates show that more than nearly 1 billion individuals are affected by OSA worldwide [3]. In the middle-aged labor force, approximately 4% of men and 2% of women are likely to fulfill the minimal diagnostic standards for the OSA syndrome [4]. Usually, patients with OSA have loud snoring [5] and are suffering from a range of harmful sequelae, including daytime fatigue, morning headache [6], fatigue-related accidents and risk of systemic hypertension and heart failure [7]. Even though not every snoring person has sleep apnea, Huang et al. [8] estimates that 10% of habitual snorers are at a high risk of complete airway collapse which leads to OSA.

The typical phenomenology of OSA concerns the soft palate. The human palate forms a separation between the nasal and oral cavities. It is comprised of two distinct parts, i.e., hard palate and soft palate [9]. The hard palate is immobile, consisted of bone, while the soft palate is a movable soft tissue, suspended from the posterior border of the hard palate. The respiratory airflow can create instabilities and vibrations in the soft palate, resulting in snoring sound. In the case of sleep apnea, the airway is blocked due to posterior collapse of the soft palate. Another common pattern of OSA is related to pharyngeal collapse [10]. At the level of the oropharynx, the collapse is lateral, most commonly due to the presence of large tonsils. In the hypopharynx or the tongue area, the tongue base collapses antero-posteriorly and squeezes the epiglottis in between to cause complete obstruction.

Compliant or elastic structures subjected to fluid flow can exhibit strong oscillation, resulting from the interaction between the flow and the structural components. The interplay between the fluid and structural components is usually termed as Fluid Structure Interaction (FSI). FSI happens in a wide range of systems, from the flapping of bee wings, the flag flutter, the aircraft wing flutter, to the vibration of human soft palate. FSI-based modeling of OSA has gained a remarkable momentum in the recent scientific literature [11,12,13]. The main focus of such FSI analyses was the dynamics of the airflow and the soft tissues. In most of the investigations, 3D accurate, imaged models are reconstructed from Magnetic Resonance Imaging (MRI) and Computer Tomography (CT) scanning. Huang et al. [14] developed a 3D finite element model from CT image data. The model included realistic anatomical details of the airway surrounding structures such as the skull, neck, cartilage and soft tissues. FSI simulations were performed both for normal and OSA respiratory processes. The simulation results demonstrate a positive feedback of the upper airway collapse through the interaction of the anterior airway wall and the pressure gradient on the cross-section area during OSA. In [13], Pirnar et al. reestablished the pharyngeal geometric model from CT images of a patient, where the soft palate with uvula was considered as an isolated structure. They found that airway occlusion could occur at the region of the velopharynx without considering gravity during inspiratory process. Chen et al. [15] used a reconstructed 3D model of the upper airway to analyze the impact of the uvula flapping on the airway vibration. From the simulation results, they demonstrated that the flapping frequencies of the uvula tend to affect more significantly on the airway vibration compared with the flapping amplitude. However, in their work, the uvula was considered to be a rigid body with certain flapping frequencies. And the airflow was assumed to be incompressible.

Due to high computational cost and geometric complexity of the 3D anatomically accurate model, several studies were conducted on a simplified model of the upper airway. The main advantage of employing simpler geometries is that the fundamental physical behaviour of the system clearly emerges and can be more easily controlled [16]. Moreover, as numerical models require validation, it is much simpler to find in literature high-quality experimental data sets to validate the numerical results [17]. Tetlow & Lucey [18] modelled the soft palate as a cantilevered flexible plate placed within a unsteady, viscous, incompressible channel flow to mimic the mechanical process for the dynamics of the soft palate in OSA. They revealed that constant inlet velocity conditions can over-estimate the energy exchange between the flow and the structure. They also showed that offsetting the position of the plate within the channel can cause a larger deformation of the plate. The main limitation of the study was that the model was 2D and the plate was treated as a classical thin plate, which means the transverse shear stress was out of consideration. Viscous effects on the plate were also ignored. In order to have a better comprehension of the OSA mechanisms, Khalili et al. [19] developed a new 2D model of the upper airway. The soft palate was modeled as a flexible plate, placed in a compressible, viscous, laminar channel flow. The flow patterns were analyzed when both the nasal and oral inlets were open and when one of them was closed. Besides, an acoustic analysis was performed to the acoustic wave propagation induced by the palatal flutter. However, the 3D effects of the geometry were not considered. Geometry dimensions, as well as material properties (e.g., Young’s modulus, Poisson’s ratio) were not in physiological condition. Based on a 2D FSI model, Cisonni et al. [20] developed a 2D FSI model, based on a flexible, tapered cantilever axially mounted in a channel flow, to represent the uvuloplatal system in snoring conditions. The effects of material inhomogeneity on the vibrational dynamics were investigated by varying the ratio of the uvula thickness to the hard palate thickness. They demonstrated that higher degree of tapering would decrease the fluid speed required to initiate the structural vibration instability. In their work, the flow was treated incompressible in laminar regime. The flexible palate was described by the one-dimensional Kirchoff–Love beam, where the friction force was not incorporated.

The large part of the studies on the fundamental dynamical behaviour of the uvulopalatal system under OSA conditions is limited by either low dimensional studies or by strict assumptions on the airflow. In this paper, these limitations are tackled by the implementation of a simplified 3D soft palate with uvula under the effects of a compressible, viscous unsteady airflow. The flow is confined in a cylindrical tube, representing the pharyngeal airway. The effects of different inlet boundary conditions of the flow on the oscillatory behavior of the uvulopalatal system are investigated by means of a two-way FSI coupling approach. Understanding the dynamics of the uvula is preparatory to future aeroacoustic simulations that will be used for assessment of sound generation into relation with OSA.

The paper is structured as follows. In Section 2, the model development for the fluid and solid domains are described. In Section 3, the results of the fluid simulations, with the solid structure fixed, are presented. The eigen modes of the 3D solid structure are analysed by employing a modal analysis. The results of 2-way FSI simulations are finally described. An in-depth discussion of the results and conclusions with possible future developments can be found in Section 4.

## 2. Methods

In this work, a fully two-way FSI coupling [21] is employed. Within each inner time iteration, the results of the Computational Fluid Dynamics (CFD) analysis i.e., velocity, pressure, and wall shear stress at the interfaces are used to calculate the external loads imposed by the fluid on the soft structure. The consequent displacement of the solid structure is computed and transferred back to the CFD solver by morphing the fluid mesh. The geometry under analysis in this study is a simplified 3D geometry of the pharyngeal airway with a elastic structure resembling the soft palate described in Section 2.1. The implementation of the computation model used for the fluid and solid domains is reported in Section 2.2 and Section 2.3, respectively. Convergence study and numerical validation of the grid are presented at the end of this section.

### 2.1. Simplified 3D Model for Palatal System

The goal of the numerical study is to analyse a simplified 3D model of the palatal system to clarify the main physical mechanisms underlying the palatal snoring. Since the palatal system has very complex geometry and in-vivo measurements are usually not practical, with a simplified geometry it is easier to analyse the effects of vibrations of the soft palate. Moreover, simplified systems can be more easily adapted to entail the wide varying spectrum of geometries of the soft palates reported for human beings. The mean and standard deviation of the human palatal system’s geometrical dimensions and mechanical properties are reported in Table 1 and in Table 2, respectively. It is to be noted that the value of the Poisson’s ratio is connected to the material characteristics of the soft palate, which has been experimentally determined. The geometry employed in this work refers to the mean values of these tables.

**Figure 1:**
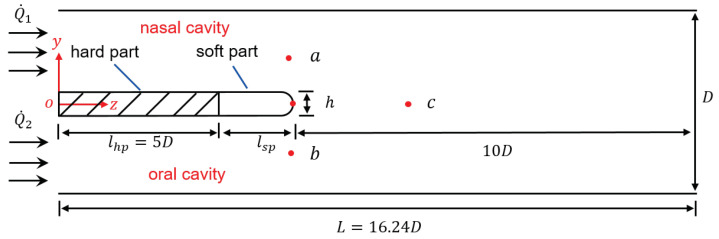
Sketch of the fluid domain with dimensions and the applied inflow boundary conditions shown.

Figure 1 shows the model geometry where the pharynx is modeled as a tube with circular cross-section. The palate system is placed in the middle of the tube, separating the nasal (upper) and oral (lower) cavities. The soft palate is attached to the back of the hard palate. The inlets are placed 5D upstream of the soft palate, while the outlet 10D downstream, to avoid spurious effects from the boundaries. The total length of the tube is 16.24D. The inlets boundary conditions can be tuned with different flow rates which correspond to different breathing conditions. In *nose-only* breathing condition, ${\dot{Q}}_{1}\neq 0,{\dot{Q}}_{2}=0$. In *symmetric* breathing condition, ${\dot{Q}}_{1}={\dot{Q}}_{2}$. A total of 6 different breathing conditions, spanning from *symmetric* to *nose-only*, are considered in this work. In the schematic presented in Figure 1, one point probe is placed on the tip of the soft palate and is used to monitor the solid displacement. The two monitoring points *a* and *b* are symmetrically placed along the vertical direction, in correspondence of the tip of the soft palate. The point probe *c* is located 3D downstream of the soft part for measuring the fluctuation in the wake region.

**Figure 2:**
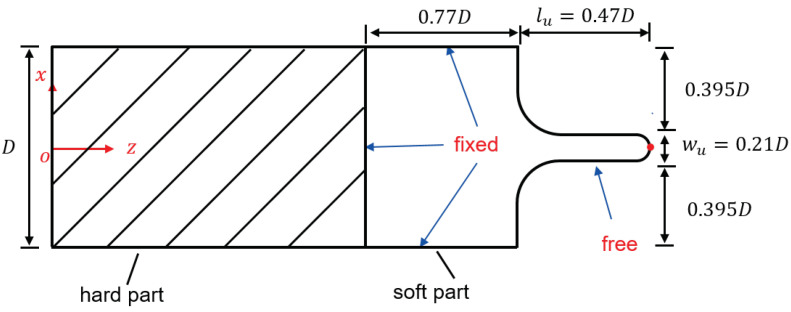
Model geometry for the soft palate with dimensions and the associated boundary conditions.

The oropharyngeal anatomy [27] shows the uvula is a drop-shaped muscular tissue attached to the posterior edge of the soft palate. The soft palate is modelled as a rectangular thick plate in Figure 2, smoothly attached by the quasi-cylindrical uvula at the middle of the side edge. All the other sides are considered clamped.

### 2.2. Fluid Domain

In the present study, the unsteady compressible 3D Navier-Stokes equations are solved to describe the fluid flow around the human palatal system. The corresponding numerical model is implemented in the commercial software Star-CCM+ (v. 17.06.008) by Siemens. The working fluid is air and it is modeled as an ideal, Newtonian, and isothermal fluid. Large Eddy Simulation (LES) is used to produce high-fidelity simulations of the instantaneous time behaviour of the system by employing less computational resources of a direct numerical simulation. Concerning the human upper respiratory tract, LES have been already successfully employed in several studies, among which the first ever LES applied to OSA was conducted by Mihaescu et al. (2011) [28]. Chen & Gutmark [29] show that LES can give better prediction of the root mean square (RMS) than the Reynolds stress model (RSM). The sub-grid scale model used in this study is the Wall-Adapting Local Eddy-viscosity (WALE) by Nicoud & Ducros [30], due to its flexibility in handling wall bounded flows and the transition regime with a reasonable computational cost. The model is closed by adopting the k−ω Shear Stress Transport (SST) [31] turbulence model. Since the Mach number of the involved flow in this study is way below 0.3, a pressure-based segregated solver is implemented, so that the conservation equations of mass and momentum are solved in a sequential manner. Despite the low Mach number, the fluid is modeled as compressible. This is motivated by the perspective to employ the current numerical model to directly assess the sound generation due to the structural vibrations [32]. This can be an additional information to be used for quantifying obstructive airway disorder. The spatial mesh grid spacing δ is tuned such that δ is larger than the Kolmogorov scale but smaller than the Taylor micro-scale. For the time integration procedure, a second-order implicit method is applied and the time step size Δt is chosen to satisfy the Courant-Friedrichs-Levy (CFL) criterion, i.e., CFL<1.0.

Flow rate boundary conditions are applied to the two inlets, while the outlet is set as zero pressure boundary. No-slip boundary conditions are imposed on the walls and at the FSI interface. In [33], experimental measurements show that in a human nasal cavity the respiration rates corresponding to calm, medium and intensive breathing are equivalent to 180 mL/s, 560 mL/s and 1100 mL/s, respectively. Table 3 shows the flow rate conditions accordingly employed in this work, where $\dot{Q}$ is the total flow rate, defined as the convex sum of the nasal cavity (${\dot{Q}}_{1}$) and the oral cavity (${\dot{Q}}_{2}$). Consequently, ${\dot{Q}}_{1}$ and ${\dot{Q}}_{2}$ can be defined in terms of the openness factor α as:

(1) $$\begin{array}{cc} {\dot{Q}}_{1} & =\alpha \dot{Q} \end{array}$$

(2) $$\begin{array}{cc} {\dot{Q}}_{2} & =\left(1-\alpha \right)\dot{Q}, \end{array}$$

where α ranges from 0.5 to 1.0. The case α=0.5 corresponds to symmetric breathing scenario (mouth-nose breathing), while α=1.0 refers to nose-only breathing with mouth closed. In this work, the FSI behaviour of the system is investigated by considering, for each flow rate in Table 3, 6 different values of the openness factor α.

### 2.3. Solid Domain

The solid domain is modeled as an isotropic linear elastic material with Young’s modulus *E*, Poisson’s ratio $\nu_{sp}$ and density $\rho_{sp}$ (see Table 2 for the corresponding numerical values). In the model, gravity is not considered. Finite Element Method (FEM) is applied to account for the structural deformation as response to the shear stress and pressure imposed by the fluid on the fluid-structure interface.

### 2.4. Mesh Sensitivity Study

The determination of an efficient mesh for both the solid and fluid domains is achieved by means of a mesh sensitivity study. Unstructured polyhedral mesh is applied to the fluid domain. The viscous sub-layer is resolved by adopting 8 prism layers on the walls. Unstructured tetrahedral mesh has been employed for the solid domain. To prevent self-locking i.e., a numerical underestimation of the displacement which can be caused by linear tetrahedral finite element, 10-node quadratic tetrahedral finite elements are employed.

**Figure 3:**
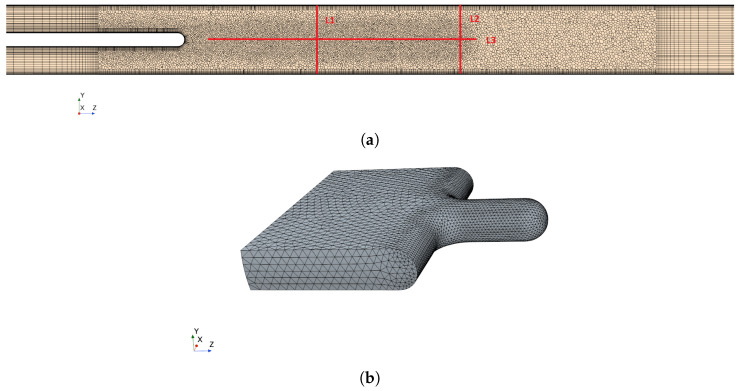
(**a**) A cut plane (x=0) of the fluid mesh, showing the locations of the velocity line probes, with $L_{1}$, $L_{2}$ cross the flow and $L_{3}$ in the axial direction. In the same region it is possible to see the mesh refinement applied in the area downstream the structure. (**b**) Mesh of the solid domain.

Figure 3a shows the topology of the fluid computational domain. The region surrounding the soft structure, where the most interesting dynamical phenomena happen, is discretized with a finer mesh which is able to resolve the FSI behaviour of the system with high fidelity. A local refinement is applied in the wake region, as is shown in Figure 3a, in order to well resolve the flow characteristics. The mesh for the solid soft structure is shown in Figure 3b. The mesh convergence study is conducted in sequential steps. The first step consists in keeping the solid structure fixed and applying three meshes of 0.6 M, 0.9 M, 1.2 M cells to the flow domain. The inflow boundary condition is set as $\dot{Q}=0.36\;L/s$, α=1.0, which corresponds to nose-only calm respiratory. Convergence is assessed by considering time-averaged axial velocity profiles on the line probes shown in Figure 3a. In the second step, the most efficient mesh is implemented for the fluid domain, and three meshes of 0.053 M, 0.3 M, 0.5 M cells are considered for the solid domain which is now allowed to deform. The time histories of the displacement magnitude on the tip point of the structure are monitored and compared to identify the most efficient solid mesh.

**Figure 4:**
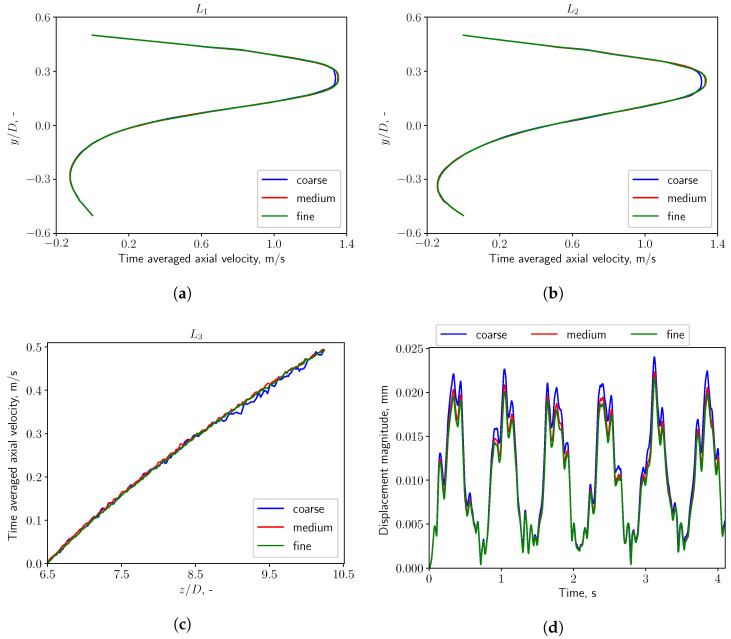
(**a**–**c**): Time-averaged axial velocity evaluated at line probes $L_{1}$, $L_{2}$ and $L_{3}$ when the solid structure is fixed. y/D, z/D: the non-dimensional *y* and *z* coordinate, normalized by the tube diameter *D*, respectively. (**d**): Time history of the displacement magnitude of the uvula tip.

As shown in Figure 4a–c, the aforementioned 3 fluid meshes with increasing resolution (noted in the figures as *coarse*, *medium*, and *fine* mesh) are applied and reach good convergence in CFD simulations. The solid grid independence study with three solid meshes is also conducted and reaches a similar result in FSI simulations, as shown in Figure 4d. The grid convergence study shows that 0.9 M fluid cells and 0.3 M solid cells are adequate for the FSI simulations.

## 3. Results

In this section, the results of the numerical simulation are presented and discussed. Section 3.1 shows the flow patterns and characteristics by analyzing the pressure fluctuation spectra in the wake region. The influence of different breathing patterns, as well as different inlet opening conditions are investigated (see Table 3). In Section 3.2, a modal analysis of the solid structure is presented to understand the vibration characteristics of the structure. When the structure is allowed to move, the solid and the fluid come to interact. The corresponding results are shown in Section 3.3.

### 3.1. Analysis of Fluid Domain

**Figure 5:**
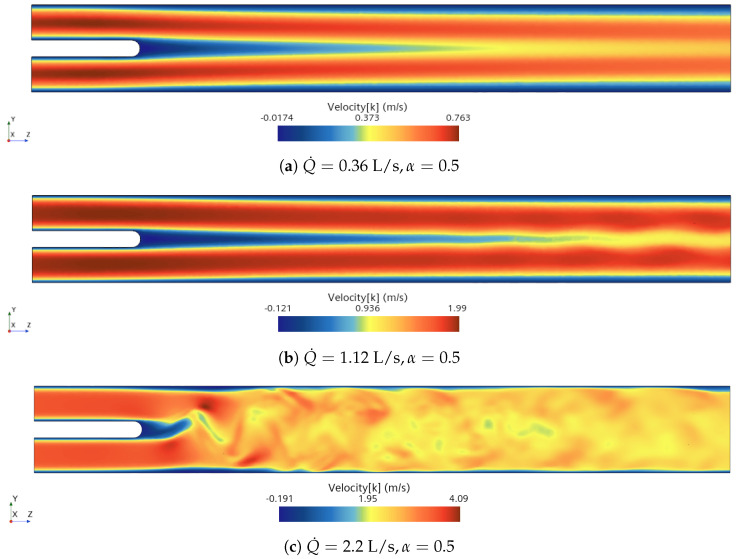
Instantaneous axial velocity fields on the *x* = 0 cut plane for three breathing flow rates with symmetric inlet boundary conditions.

The axial component of the velocity fields are presented in Figure 5. The flow of the calm breathing case ($\dot{Q}=0.36$ L/s) presents symmetric wake behind the body and no pronounced fluctuations are seen. When the boundary conditions are switched to medium breathing scenario ($\dot{Q}=1.12$ L/s), the wake starts to oscillate due to the growth of the fluctuations in the flow. When the inlet flow rate is increased to 2.2 L/s (intensive breathing), the wake is characterised by a vortex shedding from the body representing the palate system.

**Figure 6:**
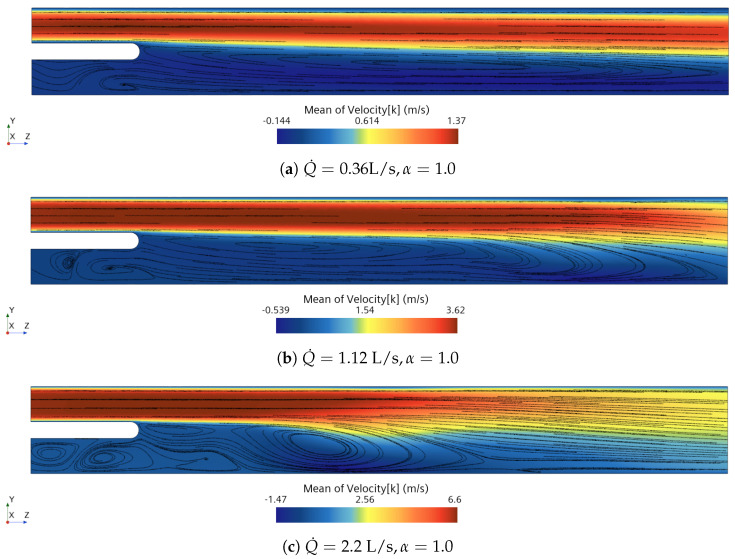
Axial velocity mean fields on the yz cut plane for three respiratory flow rates with nose-only breathing.

Figure 6 displays the mean axial flow velocity when nose-only breathing (α=1.0) occurs. In this condition, the flow coming from the nasal inlet (i.e., the upper inlet) is dominant over the oral one (lower inlet). The flow mixes downstream the body and recirculation bubbles downstream and under the body appear as described by the streamlines. Concerning the flow unsteadiness, the oscillating frequency of the flow is evaluated by analyzing the spectrum of the pressure fluctuations at the point probe located downstream the uvula shown in Figure 1. The Strouhal number is defined as:

(3) $$St=\frac{fh}{u_{mix}},$$

with *f* the oscillating frequency in the wake, *h* the height of the solid structure, and $u_{mix}$ the mixed surface-averaged velocity of the two inlets. And $u_{mix}$ is defined in the following form:

(4) $$u_{mix}=\sqrt{\alpha {\left(\frac{{\dot{Q}}_{1}}{S}\right)}^{2}+\left(1-\alpha \right){\left(\frac{{\dot{Q}}_{2}}{S}\right)}^{2}},$$

where *S* is the cross section area of one of the inlets.

**Figure 7:**
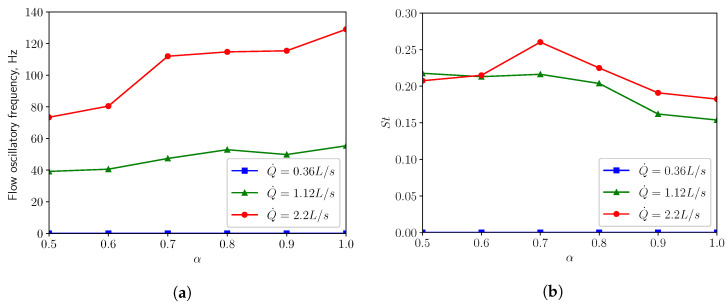
(**a**) shows the flow oscillatory frequency in the wake against the openness factor α for three different respiratory rates, with (**b**) displaying the corresponding Strouhal number.

In Figure 7a, one can see when in restful breathing condition ($\dot{Q}=0.36$ L/s), the oscillation of the flow is negligible. When the respiratory rate is increased to the medium condition ($\dot{Q}=1.12$ L/s), the flow oscillates with a frequency ranging from 40 Hz to 56 Hz. For intensive breathing condition ($\dot{Q}=2.2$ L/s), the frequency varies between 73 Hz and 130 Hz, depending on the openness of the inlets (i.e., the α parameter). Figure 7b shows that the Strouhal numbers St for both medium and intensive breathing conditions vary in the vicinity of 0.2, which is expected by the presented flow characteristics. The predicted frequencies in this study lie within the palatal snoring frequency range 21–323 Hz, which was evaluated from snoring acoustic measurements conducted by Brietzke & Mair (2006) [34]. A study by Agrawal et al. (2002) [35] revealed that patients with palatal snoring had a median peak frequency at 137 Hz.

### 3.2. Modal Analysis of Solid Structure

The purpose of modal analysis is to determine the eigenmode shapes and the corresponding natural frequencies of the solid structure during free vibration. The distribution of energy in the set of eigenmodes is not constant and often only few modes are needed to represent the motion of a structure with an acceptable approximation. In the present study, the modal analysis is carried out by employing the commercial FEM solver COMSOL Multiphysics (v6.1). The same clamped boundary conditions are applied to the three side edges, while the other edge is set free, as is shown in Figure 2.

**Figure 8:**
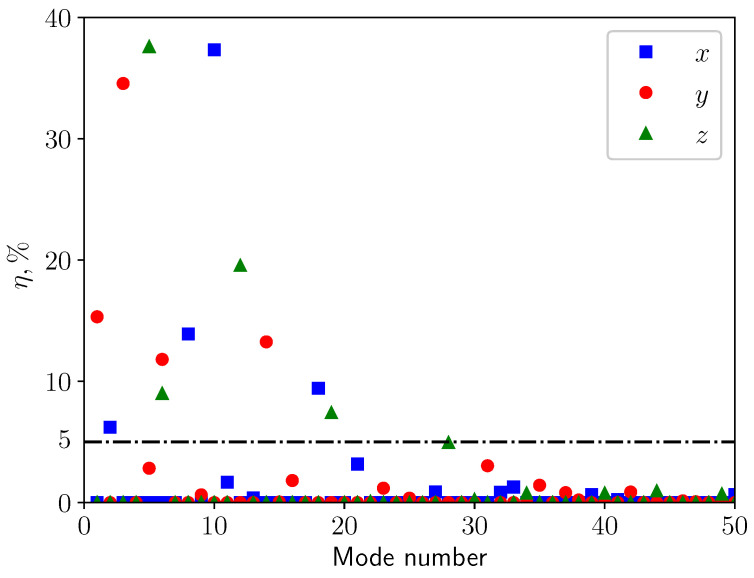
Effective mass ratio.

**Figure 9:**
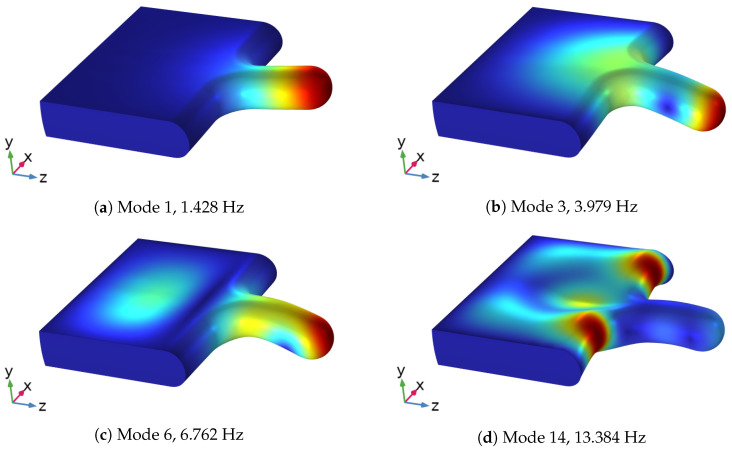
The 4 most significant modes for the vertical deformation of the structure. Color represents the displacement magnitude.

To determine which modes are significant for the representation of the response of the structure when subjected to external loads, the effective mass associated with each mode is calculated. It measures the amount of the total mass participating in that mode in a given excitation direction. A mode with large effective mass is a significant contributor to the structural response in a given excitation direction. Figure 8 shows the ratio of effective mass to the total mass of the solid η in percentage plotted against the first 50 modes for the three main directions. A threshold value of η>5% has been selected to define the most significant modes. Moreover, as will be discussed in Section 3.3, the vertical displacement (*y* direction) is dominant under most of the boundary conditions. Consequently, the analysis will focus only on the modes concerning vertical displacement (red dots in Figure 8). Modes 1, 3, 6, and 14 are the most significant modes for the vertical deformation, as can be seen in Table 4. Their mode shapes are presented in Figure 9. Mode 1 is a simply bending mode, while the other three are higher-order modes. Mode 14, the lateral (in the *x*-direction) part of the soft palate undergoes larger deformation compared with the uvula’s tip, whereas the other three modes have larger deformation on the tip point.

### 3.3. FSI Simulations

**Figure 10:**
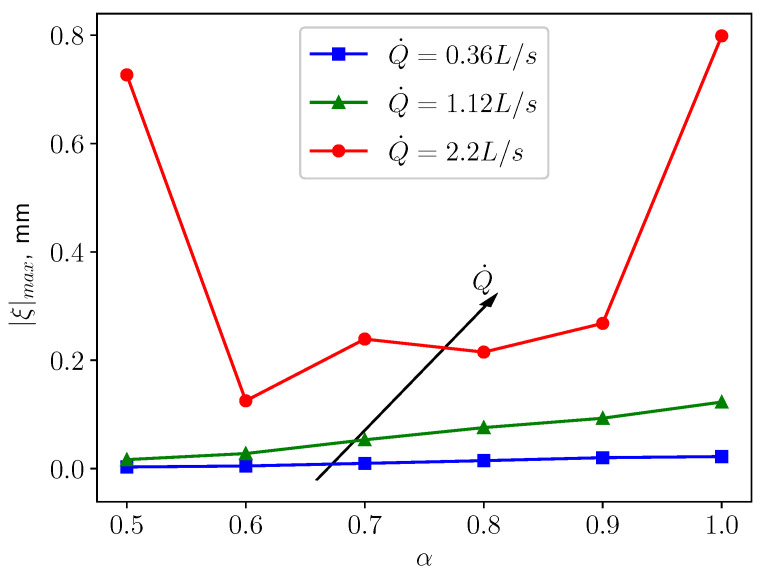
The maximum tip displacement magnitude of the structure under different respiratory rates.

In fully coupled FSI scenario, the soft palate starts to vibrate due to the interaction with the flow. The pressure and the wall shear stress imposed by the fluid are driving the motion of the soft palate. By following the same boundary conditions scheme of the previous section, the simulation’s goal is to investigate the effects of the three breathing regimes on the motion of the structure. In particular, the displacement on the tip point of the structure (see Figure 2) is monitored. Figure 10 shows the maximum displacement magnitude of the tip point. The displacement magnitude is defined as

(5) $$\xi =\sqrt{\xi_{x}^{2}+\xi_{y}^{2}+\xi_{z}^{2}},$$

with $\xi_{x}$, $\xi_{y}$, $\xi_{z}$ the displacement in *x*, *y* and *z* direction, respectively. The maximum displacement magnitude indicates the largest deformation that the structure can have and it is employed as a kinematic representative of the dynamics of the solid.

**Figure 11:**
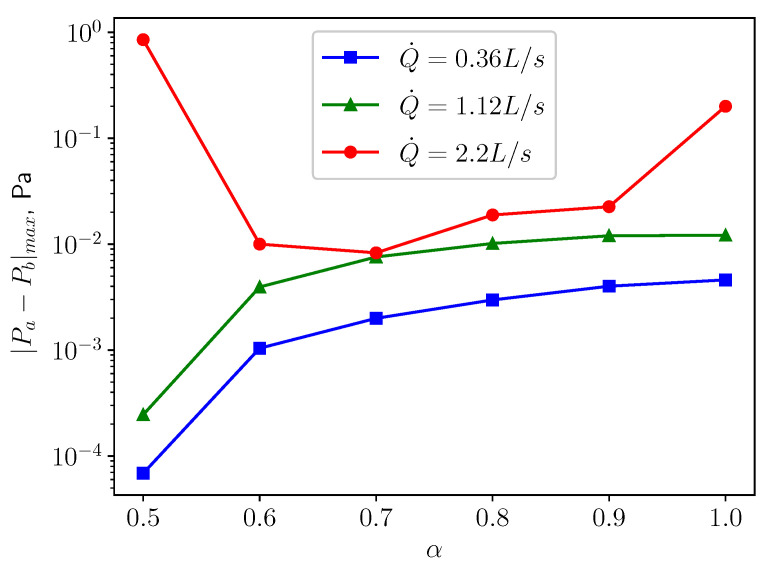
The maximum pressure difference between point probes *a* and *b* (see Figure 1) located above the top and below the bottom surfaces of the uvula, respectively.

Figure 10 shows that as $\dot{Q}$ grows, the maximum displacement magnitude ${\left|\xi \right|}_{max}$ increases. This can be expected since the fluid will exert larger pressure and wall shear stress on the structure, leading to larger deformation. A similar pattern is displayed in Figure 11, which shows that higher respiratory rate results in larger pressure difference between the top and bottom surfaces of the uvula, which causes to larger deformation. For the cases of calm ($\dot{Q}=0.36$ L/s) and medium ($\dot{Q}=1.12$ L/s) breathing, the structure undergoes larger displacement as the openness factor α increases. One possible explanation is that the flow from the nasal inlet is dominant over the flow coming from the oral inlet. Consequently, the pressure difference acting on the structure increases, as is shown in Figure 11, causing larger deformation in the structure. However, in the case of intensive respiration, ${\left|\xi \right|}_{max}$ and α are not in a monotonically increasing relation anymore. The structure undergoes large deformation when the inlet boundary conditions are symmetric (α=0.5). The displacement decreases quickly as α increases from 0.5 to 0.6. For larger values of α, the displacement increases again almost in a monotonic fashion. When α reaches 0.9, a quick increment in the deformation emerges. This behavior is probably because more fluctuations and small structures in the flow are generated and provide more energy to the vibration of the soft palate. In the next subsections, a careful analysis of the behaviour of the soft palate under different breathing conditions is provided. In particular, to have a better understanding of the underlying physical mechanisms, the displacement spectra of the structure will be analysed.

#### 3.3.1. Calm Breathing: $\dot{Q}=0.36$ L/s

**Figure 12:**
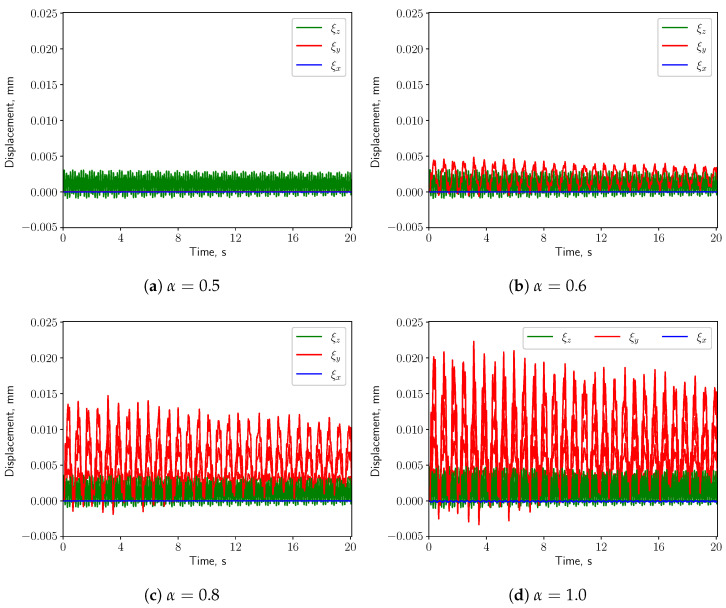
(**a**–**d**) show the displacement components at the structure tip when $\dot{Q}=0.36$ L/s.

For the calm respiratory scenario, the time histories of the displacement components of the uvula’s tip are shown in Figure 12, for different values of α. It can be seen that higher values of α correspond to a larger vertical displacement ($\xi_{y}$). The amplitude of the span-wise displacement ($\xi_{x}$) is almost constant w.r.t. α, while the axial displacement ($\xi_{z}$) amplitude slightly increases. Moreover, $\xi_{y}$ is dominant over the other two components when the inlet boundary conditions are not symmetric, i.e., α≠0.5. The pressure difference between the top and the bottom surfaces of the solid structure grows, causing larger vertical deformation. For this reason, the following analysis concerns only the vertical displacement.

**Figure 13:**
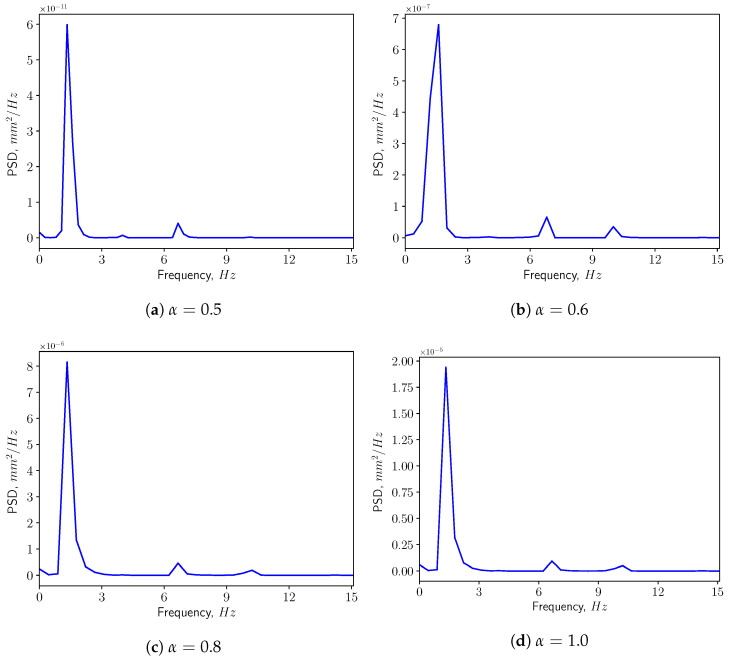
Power spectrum density of the vertical displacement ($\xi_{y}$) at the tip in calm breathing ($\dot{Q}=0.36$ L/s).

**Figure 14:**
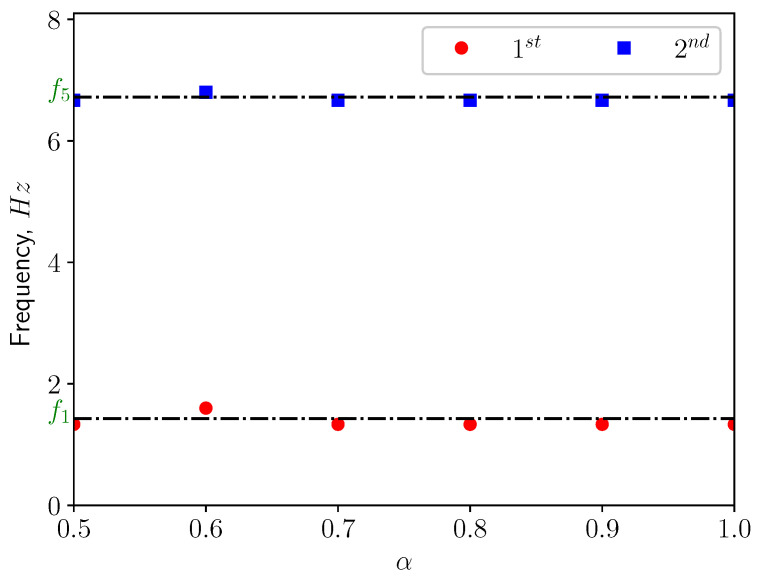
The first two dominant frequencies of the power spectrum of the vertical displacement ($\xi_{y}$) in calm breathing ($\dot{Q}=0.36$ L/s).

Figure 13 presents the corresponding spectra of the vertical displacement ($\xi_{y}$), with the Power Spectrum Density (PSD) plotted versus the frequency. The first two dominant frequencies are compared with the natural frequencies of the solid structure in Figure 14, with $f_{1}$ and $f_{5}$ corresponding to the first and the fifth eigen-frequencies of the solid domain (see Table 4). From the modal analysis in Section 3.2, the larger contribution to the vertical deformation comes from the first mode. The power level of the first peak increases as α increases, as can be seen in the spectrum. With higher openness factor α, the larger difference in pressure between the two sides of the solid induces larger deformation in the soft palate in the vertical direction.

**Figure 15:**
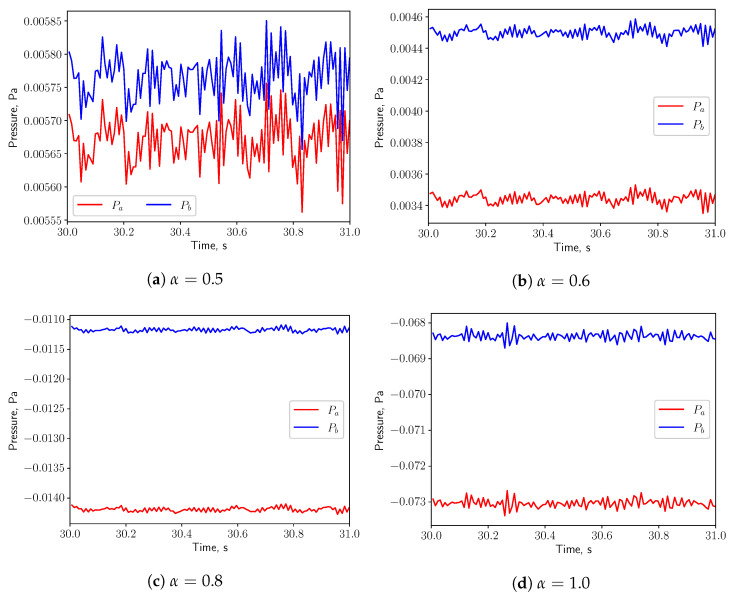
(**a**–**d**) show the pressure signals on the top and bottom surface of the uvula in calm breathing ($\dot{Q}=0.36$ L/s).

Figure 15 shows the time history of the pressure at the probe points *a* and *b* shown in Figure 1 on the top and bottom of the uvula. Both pressure signals are in phase, indicating that the effect of the vortex shedding are negligible under such low respiratory rate. Since the fluid flow is not oscillating, no resonance effects between the solid and the fluid are expected (see Figure 7a).

#### 3.3.2. Medium Breathing: $\dot{Q}=1.12$ L/s

**Figure 16:**
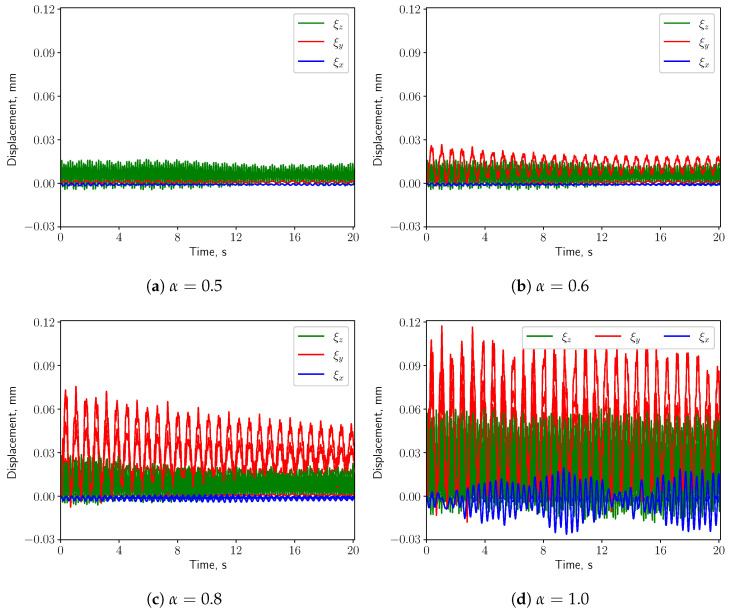
(**a**–**d**) show the displacement components at the structure tip when $\dot{Q}=1.12$ L/s.

When the medium respiratory rate boundary conditions are employed, the solid structure undergoes larger deformation compared with the calm breathing cases. Remarkably, also the span-wise and axial components of the displacement shown in Figure 16 increase as α grows. This is mainly because the pressure gradients in vertical and span-wise directions as well as the drag force acting on the solid structure are becoming larger, compared with the calm breathing cases. When α=0.5, due to the symmetry of the inlet boundary conditions and of the geometry, the small pressure differences in *x* & *y* directions induce small deformation in these two directions w.r.t. the vertical direction. The axial deformation is dominant, which is mainly driven by the drag force imposed by the fluid. As long as the inlet boundary conditions are no more symmetric, i.e., α≠0.5, the vertical deformation starts to increase rapidly w.r.t. the other ones.

**Figure 17:**
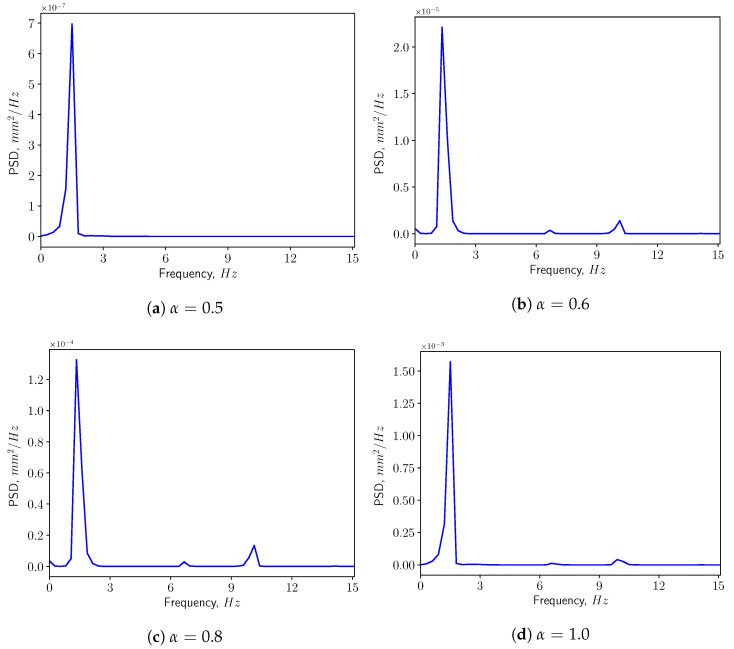
Power spectrum density of the vertical displacement ($\xi_{y}$) at the tip in medium breathing ($\dot{Q}=1.12$ L/s).

**Figure 18:**
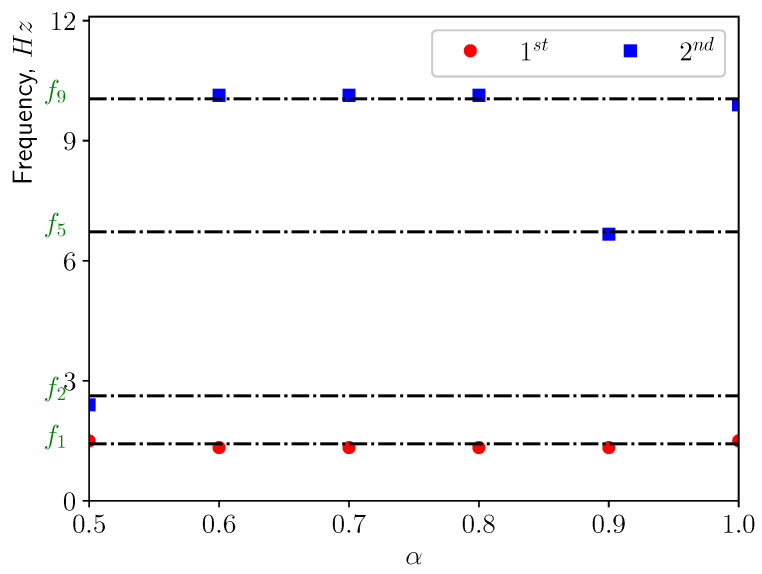
The first two dominant frequencies of the power spectrum of the vertical displacement ($\xi_{y}$) in medium breathing ($\dot{Q}=1.12$ L/s).

The power spectrum of the solid displacement $\xi_{y}$ is computed and presented in Figure 17. The first two dominant frequencies are shown in Figure 18. The frequency corresponding to the first peak coincides with the first eigen-frequency of the structure. This means that under this conditions, the first eigen-mode of the structure is excited. This can be confirmed by Figure 18, where $f_{2}$, $f_{5}$ and $f_{9}$ are the natural frequencies of the eigen-modes 2, 5, and 9. From Table 4, one can see the contributions from modes 2, 5, and 9 are less than 5%. Additionally, it can be noted that the power level of the first peak increases as α grows since the amplitude of $\xi_{y}$ is larger.

**Figure 19:**
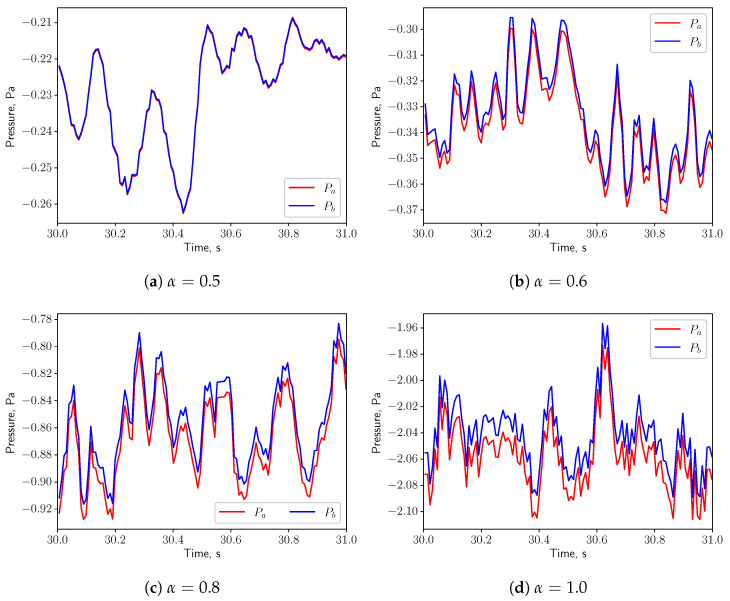
(**a**–**d**) show the pressure signals on the top and bottom surface of the uvula in medium breathing ($\dot{Q}=1.12$ L/s).

The pressures at the probe points shown in Figure 19 oscillate in phase for all the values of α, indicating that the effects of the vortex shedding are negligible under this breathing scenarios. Resonance is not expected, since the natural frequency of the solid (1.428 Hz) is not matching from the flow oscillatory frequency (see Figure 7a).

#### 3.3.3. Intensive Breathing: $\dot{Q}=2.2$ L/s

**Figure 20:**
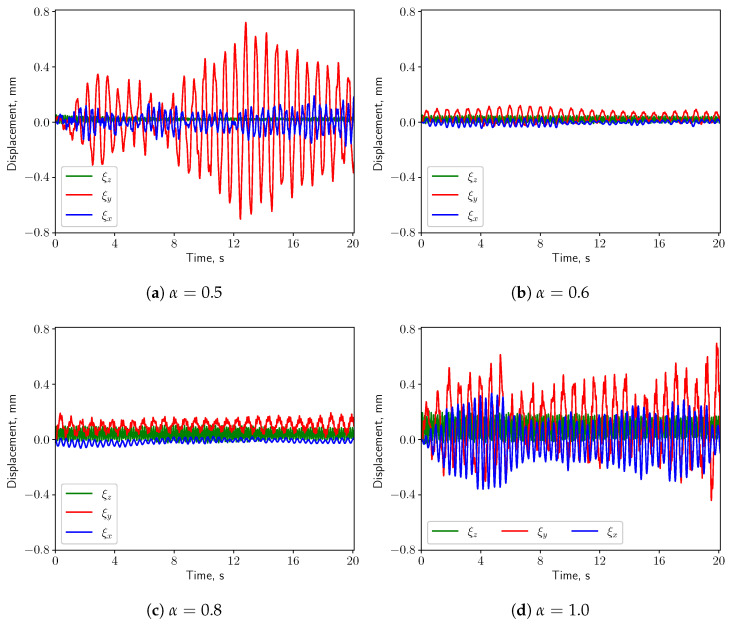
(**a**–**d**) show the displacement components at the structure tip when $\dot{Q}=2.2$ L/s.

When intensive breathing rate is implemented at the inlet boundaries, the deformation of the structure is larger (see Figure 20). Interestingly, under symmetric condition (α=0.5) the structure undergoes comparable deformation as the case of nose-only breathing (α=1.0). The displacements decrease as α grows from 0.5 to 0.6, and then increase again for larger values of α. Such behavior is remarkably different than the calm and medium breathing cases. Figure 5 shows a vortex which is shed from the soft palate, whereas in the other two cases no pronounced vortex shedding appears. The large deformation in Figure 20a is connected to the vortex shedding, which induces a phase shift in the pressure signal on both sides of the soft palate.

**Figure 21:**
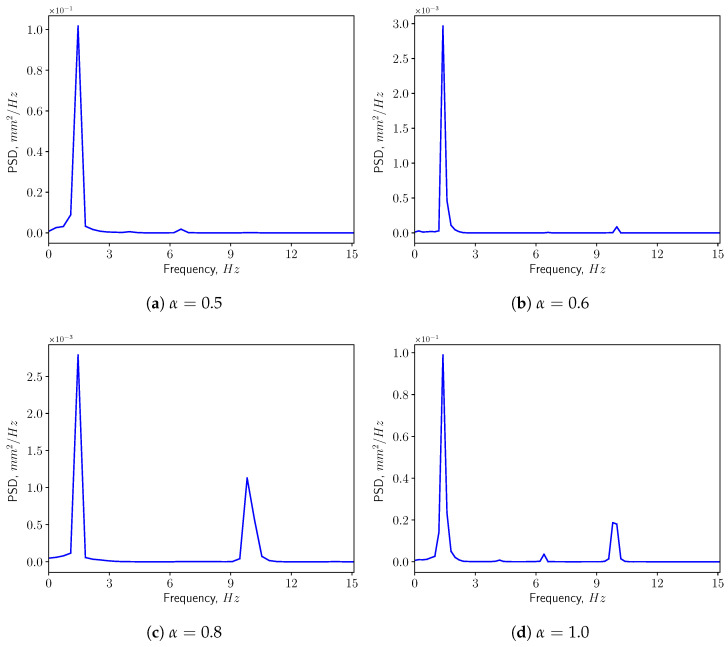
Power spectrum density of the vertical displacement ($\xi_{y}$) at the tip in intensive breathing ($\dot{Q}=2.2$ L/s).

**Figure 22:**
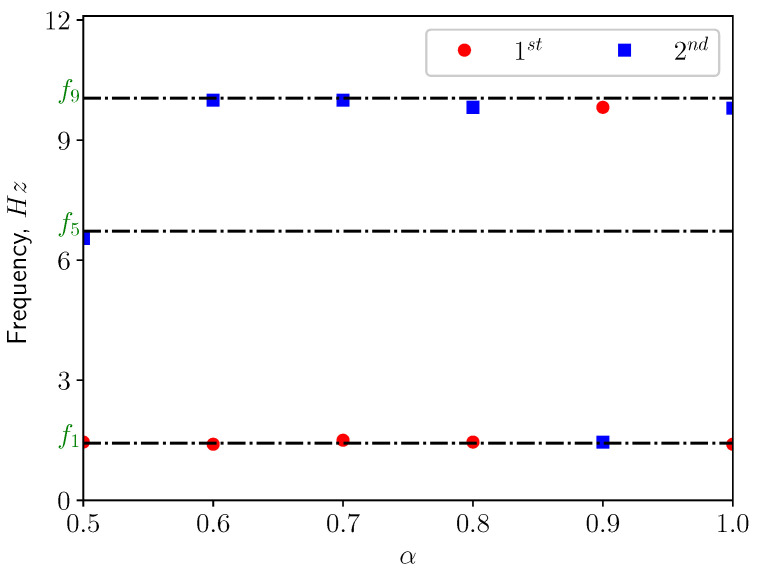
The first two dominant frequencies of the power spectrum of the vertical displacement ($\xi_{y}$) in intensive breathing ($\dot{Q}=2.2$ L/s).

Figure 21 shows the power spectrum density of the vertical displacement of the solid structure. The first two dominant frequencies for each case are presented in Figure 22, comparing with the eigen-frequencies of the structure. In the spectra, one can see that for most of the cases the first dominant frequency still coincides with the first natural frequency of the solid structure. This means that the first eigen-mode of the structure plays significant role in the vertical bending of the soft palate. For the case with α=0.9, the first dominant frequency is close to the 9th eigen-frequency of the solid. From Table 4, it is possible to remark that the effective mass ratio associated to this mode is less than 1%. Consequently, it is possible to neglect the contribution of this mode on the displacement.

**Figure 23:**
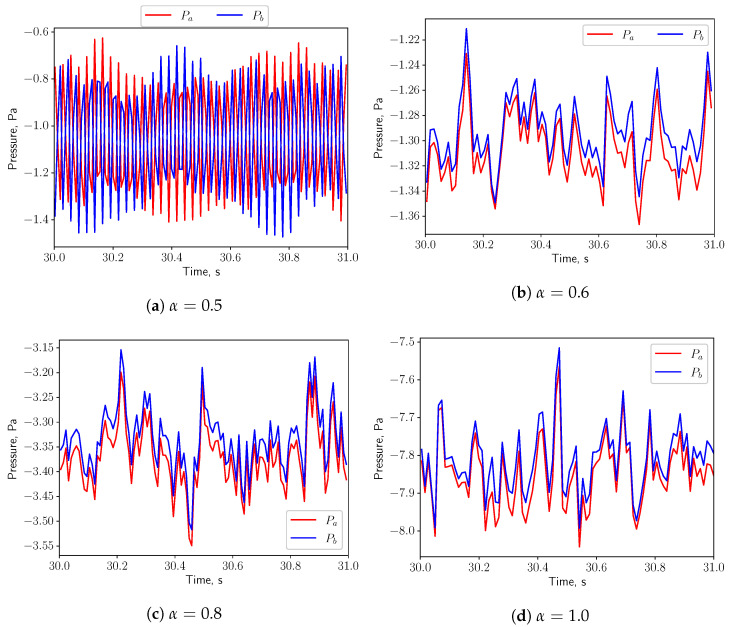
(**a**–**d**) show the pressure signals on the top and bottom surface of the uvula in intensive breathing ($\dot{Q}=2.2$ L/s).

In Figure 23, for symmetric breathing condition (α=0.5), the time histories of the pressure at the probe points on the top and bottom of the uvula are presented. It is possible to remark that the pressure signals have a 180deg phase shift, which is associated to the vortex shedding. High pressure and low pressure zones appear alternately on the both sides of the solid structure. This gives a positive feedback on the structure and eventually leads to larger deformation. When α is increased to 0.6, the flow is no more in a symmetric condition and the pressures on the top and bottom surfaces of the structure oscillate in phase. No positive feedback from the flow is added to the solid structure and the displacement decreases. Even though the vortex shedding process is present in the symmetric case and the vibration of the solid structure is intensified, no resonance effects are expected, as the natural frequency of the solid (1.428 Hz) does not match with the vortex shedding frequency (see Figure 7a).

## 4. Discussion & Conclusions

A simplified 3D airway model including the soft palate in physiological boundary and geometric conditions is developed to study the interaction among the uvulopalatal system and the airflow by employing two-way coupling FSI analysis. The vibrational response of the soft structure is found to be considerably dependent on the investigated parameters i.e., the inlet flow rate $\dot{Q}$ and the inlet openness factor α.

The present study’s simplified 3D airway model makes it possible to investigate the uvulopalatal vibrational characteristics and the airflow changes. The FSI analysis shows that larger structural deformations are present for increasing inlet respiratory rate $\dot{Q}$. In the cases of calm and medium breathing scenarios, the structure undergoes larger displacements for larger values of the openness factor α. In intensive respiration, the structure undergoes large deformations for α=0.5. This is mainly due to the positive feedback from the vortex shedding of the flow. However, the resonance between the structure and the fluid does not occur since the natural frequency and the flow oscillation frequency do not match. The effects of the axial velocity on the components of the structural displacement can be investigated by analyzing the results from the analysis of the volume flow rate $\dot{Q}$. When $\dot{Q}$ is increasing, as can be seen from Figure 12, Figure 16 and Figure 20, *y* displacement (vertical) and *z* displacement (axial) grow accordingly.

As is shown in Figure 20a, from a phenomenological standpoint, the vortex-induced vibration is not associated to a linear resonance. The 3D FSI description provided in this work, is able to link the more complex vortex shedding phenomenology to the motion of the soft palate. The 3D description allows for an in-depth description of the coupling between the airflow and the soft palate. Such understanding is crucial to determine the behaviour of the soft palate under pathological conditions. As the factor α increases, the pressures on the top and bottom surfaces of the structure are oscillating in phase due to the non-symmetric boundary conditions. For 0.6<α<0.9, the deformation increases almost monotonically. There is a sharp increment when α varies from 0.9 to 1.0. This is probably because more fluctuations and small structures in the flow are generated and provide more energy to the solid structural vibration.

The solver used in this paper has been previously validated using available experimental data for different simulated scenarios. The chosen numerical flow solver setup has been validated in a previous study by comparing the predicted surface pressures resulting from the compressible flow solution through a vocal folds model against corresponding experimental pressure wall measurements [32]. In a different study which considered changes in the convergent-divergent vocal tract model geometry (using different trans-glottal angles), the compressible flow solver has been validated by comparing the pressure predictions to corresponding available experimental mid-line pressure data [36]. Concerning the abilities of the current solver to predict deformations, the buckling critical pressure of a collapsible tube as a function of cross-sectional area—the “tube law”—has been obtained and validated against experimental data for different geometrical parameters associated with the flexible tube [16]. In all these cases, an overall good agreement between the pressure values predicted by the solver and the experimental pressure measurements has been reached.

One of the limitations of this study is that gravity and damping effects are not included in the modelling scheme. The effect of these assumptions on the structural response will be object of a future investigation. Another limitation is that the solid structure is assumed to be linear elastic. Figure 10 shows that the largest deformation under physiological conditions can be as high as 11% compared with the height of the structure. A parametric study comparing different material laws, such as Neo-Hookean elasticity and the Ogden hyperelasticity, is to be conducted. In this work, the variability and uncertainty of the geometry and material properties of the uvulopalatal system is not investigated. A further study considering these uncertainty effects [37] is planned.

An interesting perspective to generalize the results of this work, is to consider unsteadiness in the boundary conditions, to include in the picture the natural time dependence of the human respiratory rate. Another future work is to study the effects of the pharyngeal airway occlusion on the dynamics of the uvulopalatal system.

## Figures and Tables

**Table 1:** Anatomical dimensions of the soft palate.

	Values from Literature	Values Employed in This Study
Pharyngeal radius, *D* (cm)	3.8 [22]	3.8
Soft palate length, $l_{sp}$ (cm)	4.7 ± 0.7 [23]	4.7 (=1.24*D*)
Palate thickness, *h* (cm)	0.74 ± 0.14 [24]	0.74 (=0.19*D*)
Uvula length, $l_{u}$ (cm)	1.8 ± 0.1 [25]	1.8 (=0.47*D*)
Uvula width, $w_{u}$ (cm)	0.8 ± 0.04 [25]	0.8 (=0.21*D*)

**Table 2:** Mechanical properties of the soft palate.

	Values from Literature	Values Employed in This Study
Young’s modulus, *E* (Pa)	585–1410 [26]	600
Poisson’s ratio, $\nu_{sp}$ (-)	0.22–0.54 [26]	0.45
Density, $\rho_{sp}$ (kg/m${}^{3}$)	1022–1116 [26]	1060

**Table 3:** Various respiratory rates in a real human nose [33].

Breathing Condition	Calm	Medium	Intensive
Flow rate, **$\dot{Q}$** (L/s)	0.36	1.12	2.2

**Table 4:** The first 15 modes of the solid structure with the effective mass ratio in *y* direction.

Mode	Natural Frequency (Hz)	Effective Mass Ratio, η (%)
1	1.428	15.32
2	2.623	7.22 $\times \;10^{-8}$
3	3.979	34.56
4	6.122	2.07 $\times \;10^{-5}$
5	6.726	2.83
6	6.762	11.81
7	8.515	3.95 $\times \;10^{-10}$
8	8.712	5.75 $\times \;10^{-8}$
9	10.061	0.64
10	11.086	4.20 $\times \;10^{-8}$
11	11.090	5.47 $\times \;10^{-6}$
12	11.943	9.74 $\times \;10^{-9}$
13	13.348	1.00 $\times \;10^{-3}$
14	13.384	13.26
15	14.221	0.05

## Data Availability

Simulation and post-processed data can be provided by the corresponding author under reasonable request.

## Abbreviations

The following abbreviations are used in this manuscript:

OSA	Obstructive Sleep Apnea
OSAS	Obstructive Sleep Apnea Syndrome
FSI	Fluid Structure Interaction
CFD	Computational Fluid Dynamics
FEM	Finite Element Method
PSD	Power Spectrum Density
MRI	Magnetic Resonance Imaging
CT	Computer Tomography
